# Cloning, expression in *Pichia pastoris*, and characterization of a thermostable GH5 mannan endo-1,4-*β*-mannosidase from *Aspergillus niger *BK01

**DOI:** 10.1186/1475-2859-8-59

**Published:** 2009-11-13

**Authors:** Do Bien-Cuong, Dang Thi-Thu, Jean-Guy Berrin, Dietmar Haltrich, To Kim-Anh, Jean-Claude Sigoillot, Montarop Yamabhai

**Affiliations:** 1 Institute of Biological and Food Technology, Hanoi University of Technology, Hanoi, Vietnam; 2 INRA, UMR1163 de Biotechnologie des Champignons Filamenteux, ESIL, 163 avenue de Luminy, CP 925, 13288 Marseille Cedex 09, France; 3 BOKU - University of Natural Resources and Applied Life Sciences, Vienna, Austria; 4 Université de Provence, UMR1163 de Biotechnologie des Champignons Filamenteux, ESIL, 163 Avenue de Luminy, CP 925, 13288 Marseille Cedex 09, France; 5 School of Biotechnology, Institute of Agricultural Technology, Suranaree University of Technology, 111 University Avenue, Nakhon Ratchasima, 30000, Thailand

## Abstract

**Background:**

Mannans are key components of lignocellulose present in the hemicellulosic fraction of plant primary cell walls. Mannan endo-1,4-β-mannosidases (1,4-β-D-mannanases) catalyze the random hydrolysis of β-1,4-mannosidic linkages in the main chain of β-mannans. Biodegradation of β-mannans by the action of thermostable mannan endo-1,4-β-mannosidase offers significant technical advantages in biotechnological industrial applications, *i.e*. delignification of kraft pulps or the pretreatment of lignocellulosic biomass rich in mannan for the production of second generation biofuels, as well as for applications in oil and gas well stimulation, extraction of vegetable oils and coffee beans, and the production of value-added products such as prebiotic manno-oligosaccharides (MOS).

**Results:**

A gene encoding mannan endo-1,4-β-mannosidase or 1,4-β-D-mannan mannanohydrolase (E.C. 3.2.1.78), commonly termed β-mannanase, from *Aspergillus niger *BK01, which belongs to glycosyl hydrolase family 5 (GH5), was cloned and successfully expressed heterologously (up to 243 μg of active recombinant protein per mL) in *Pichia pastoris*. The enzyme was secreted by *P. pastoris *and could be collected from the culture supernatant. The purified enzyme appeared glycosylated as a single band on SDS-PAGE with a molecular mass of approximately 53 kDa. The recombinant β-mannanase is highly thermostable with a half-life time of approximately 56 h at 70°C and pH 4.0. The optimal temperature (10-min assay) and pH value for activity are 80°C and pH 4.5, respectively. The enzyme is not only active towards structurally different mannans but also exhibits low activity towards birchwood xylan. Apparent K_m _values of the enzyme for konjac glucomannan (low viscosity), locust bean gum galactomannan, carob galactomannan (low viscosity), and 1,4-β-D-mannan (from carob) are 0.6 mg mL^-1^, 2.0 mg mL^-1^, 2.2 mg mL^-1 ^and 1.5 mg mL^-1^, respectively, while the k_cat _values for these substrates are 215 s^-1^, 330 s^-1^, 292 s^-1 ^and 148 s^-1^, respectively. Judged from the specificity constants k_cat_/K_m_, glucomannan is the preferred substrate of the *A. niger* β -mannanase. Analysis by thin layer chromatography showed that the main product from enzymatic hydrolysis of locust bean gum is mannobiose, with only low amounts of mannotriose and higher manno-oligosaccharides formed.

**Conclusion:**

This study is the first report on the cloning and expression of a thermostable mannan endo-1,4-β-mannosidase from *A. niger *in *Pichia pastoris*. The efficient expression and ease of purification will significantly decrease the production costs of this enzyme. Taking advantage of its acidic pH optimum and high thermostability, this recombinant β-mannanase will be valuable in various biotechnological applications.

## Background

β-1,4-Mannans are among the main hemicellulose components that are widespread in wood, tubers, plant seeds and beans [1,2]. The structural diversity of mannans allows for a wide range of physico-chemical properties. When some of the mannose residues are replaced by glucose residues, as in the glucomannans, or substituted with galactose residues, as in galactomannans, the water-solubility of the polymers increases, whereas pure mannans are insoluble [3]. In lignocellulosic biomass these polysaccharides associate with lignin and cellulose, protecting the fibers against degradation by cellulases and microbial attack [1]. Lignocellulose is seen as a promising feedstock for the production of second-generation biofuels. For this application, the carbohydrate components in lignocellulose should be completely converted into ethanol, and enzymatic degradation of these polysaccharides is an attractive approach.

Thermostability of the employed enzymes is essential during the pre-treatment step in the conversion of lignocellulosics biomass to fermentable sugars, since steam is used to make the biomass more accessible to enzymatic attack. Thus, enzymatic hydrolysis can take place directly after the heating step, without the need to significantly pre-cool the system; hence shortening processing time, saving energy, lowering of risk of contamination, and improving saccharification and fermentation yields. Thus, overall economy of the process can be increased [4,5]. Furthermore, the saccharification step can be combined with the fermentation (simultaneous saccharification and fermentation, SSF) in order to reduce the inhibition of hydrolysis by glucose or other monosaccharides. Since fermentations for bioethanol will be run under aseptic conditions to reduce costs, increased fermentation temperatures will reduce the risk of microbial contamination. Therefore, increased thermostability for the saccharification enzymes is preferable.

Biodegradation of β-mannans by the action of thermostable mannan endo-1,4-β-mannosidase or 1,4-β-D-mannan mannanohydrolase (E.C. 3.2.1.78), commonly known as β-mannanase, offers significant technical advantages for delignification of kraft pulps [6] and for various industrial applications, such as oil and gas well stimulation [2,7], extraction of vegetable oils or coffee beans [6,8], bioconversions of non-utilized lignocellulosic substrates rich in mannan into added-value products (e.g., chemicals, feed, prebiotic manno-oligosaccharides) [2,9], or for the production of second generation biofuels [8-10].

Mannan endo-1,4-β-mannosidases, which are stable and efficiently functional at high temperatures, are found in several thermophilic bacteria, eubacteria [7,10-12], actinomycetes [13] and fungi [4,8,14,15]. Some genes encoding thermostable mannan endo-1,4-β-mannosidase of bacterial origin have been cloned, sequenced and expressed in *E. coli *[11,16]. Most of these enzymes belong to glycosyl hydrolase family 26 (GH26) according to the Carbohydrate Active Enzymes database, http://www.cazy.org[17]. Mannan endo-1,4-β-mannosidases that belong to glycosyl hydrolase family 5 (GH5) are found in bacteria [18], fungi [19] as well as higher plants [20]. Up to now, only one thermostable GH5 mannan endo-1,4-β-mannosidase gene from the fungus *Bispora *sp. MEY-1 was cloned and expressed in *Pichia pastoris *[15].

Our preliminary studies showed that the recently isolated strain *Aspergillus niger *BK01 (formerly termed *Aspergillus *sp. BK) produced thermostable mannan endo-1,4-β-mannosidase activity [21]. However, the high viscosity of the induction media containing guar gum and coffee pulp waste caused difficulties for fermentation and thus limited the scale-up for the production of this enzyme on an industrial scale. Molecular biology technology provides an opportunity to express and subsequently purify *A. niger *mannan endo-1,4-β-mannosidase in host strains capable of producing large amounts of secreted protein by industrial-scale fermentations based on well-established protocols [22].

This report describes cDNA cloning and successful heterologous expression of a novel thermostable GH5 mannan endo-1,4-β-mannosidase gene from *A. niger *BK01 in *P. pastoris*. In addition, several biochemical properties of the recombinant enzyme are reported.

## Results

### Cloning and sequence analysis of the mannan endo-1,4-*β*-mannosidase gene from *A. niger *BK01

**Figure 1:**
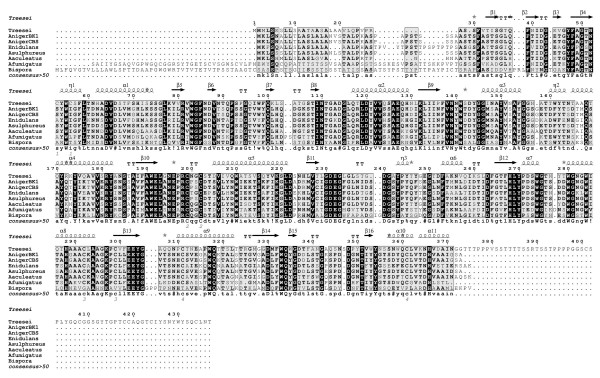
**Amino acid sequence alignment of mannan endo- β-1,4-mannosidase from *Aspergillus niger *BK01 and related fungi**. Multiple sequence alignment was done by CLUSTAL W [40] followed by ESPript [41] to display the secondary structures using *Trichoderma reesei *mannanase as a template (pdb no. 1qnr). α-Helices are displayed as squiggles; β-strands are rendered as arrows, strict β-turns as TT letters. Percent similarities of mannan endo-1,4-β-mannosidase from *A. niger *BK01 (GenBank accession no. FJ268574) to other enzymes are 51% for *T. reesei *(GenBank accession no. L25310), 99% *for A. niger *CBS 513.88 (Genbank accession no. XP_001390707), 93% for *A. sulphureus *(GenBank accession no. DQ328335), 71% for *A. aculeatus *(GenBank accession no. L35487), 66% for *Emericella nidulans *(GenBank accession no. DQ490487), 56% for *Aspergillus fumigatus *(GenBank accession no. EU925594), and 43% for *Bispora *sp. MEY-1(GenBank accession no. EU919724).

A 1035-nucleotide sequence encoding the mature mannan endo-1,4-β-mannosidase from *Aspergillus niger *BK01 (GenBank accession number FJ268574) was amplified by RT-PCR as described in the Methods section. The deduced amino acid sequence showed 70 to 93% similarity to different *Aspergillus *β-mannanases (Fig. 1). The enzyme belongs to glycosyl hydrolase family 5 [17]. It shows several characteristics common to all of the mannan endo-1,4-β-mannosidases in this family, for example a catalytic center containing catalytically/structurally important Asn, Glu, and His residues. Two catalytic glutamates (Glu196, Glu303) and five out of the six active site residues (Arg81, His129 and His268, Asn195, Tyr270, Trp333) are conserved, corresponding to the known structures of other GH5 mannan endo-1,4-β-mannosidases [13,23].

### Heterologous expression of the *A. niger *mannan endo-1,4-*β*-mannosidase in *P. pastoris*

The nucleotide sequence encoding the mature form of *A. niger *BK01 mannan endo-1,4-β-mannosidase was cloned into the pPICZαA vector. The recombinant enzyme was constructed such that the native signal peptide of the *A. niger *β-mannanase was replaced by that of the *Saccharomyces cerevisiae *α-factor signal peptide. The resulting plasmid was then transformed into *P. pastoris *by electroporation. After transformation and primary screening on plates containing Zeocin in varying concentrations, antibiotic-resistant transformants were evaluated for their ability to secrete active mannanase on plates containing Azo-carob galactomannan (BMGY-Azo). Among 40 transformants displaying mannan endo-1,4-β-mannosidase activity on the agar plates, the clone yielding the largest clearing halo was selected for cultivation in liquid culture.

**Figure 2:**
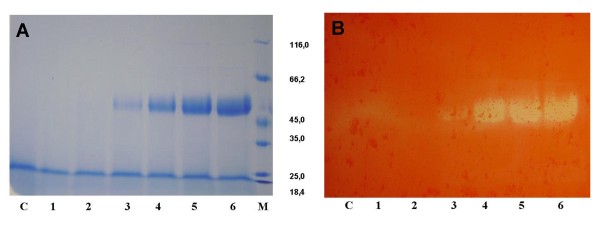
**SDS-PAGE and zymogram analysis of recombinant *A. niger *mannan endo- β-1,4-mannosidase**. SDS-PAGE (A) and zymogram (B) of secreted proteins from recombinant *Pichia pastoris *expressing the *A. niger* β -mannanase are shown. Lane M, molecular mass standard protein; Lane 1-6, culture supernatant after induction for 6, 24, 36, 48, 72 and 96 h, respectively; Lane C, control of *P. pastoris *X33 harbouring the empty pPICZαA vector after induction for 96 h. Twelve μL of culture supernatant was loaded onto each lanes.

Under appropriate conditions (2% methanol, initial cell density 6 × 10^7 ^cells mL^-1^, pH 6.0) the highest extracellular mannan endo-1,4-β-mannosidase activity (669 U mL^-1^) could be obtained after a 96-hour incubation at 28°C. No β-mannanase activity was detected in the culture medium of the control strain under identical culture conditions. SDS-PAGE analysis of the crude supernatants at the induction period from 24 to 96 h showed a single band of 53 kDa (Fig. 2A), corresponding to mannan endo-1,4-β-mannosidase activity in the zymogram analysis (Fig. 2B). The ratio of secreted to intracellular β-mannanase activity was estimated to be approximately 3:1 (data not shown).

### Purification of recombinant mannan endo-1,4-β-mannosidase

The recombinant enzyme could be purified to apparent homogeneity both by immobilized metal affinity (IMAC) chromatography and size exclusion chromatography on Superdex. However, we found that the yield of the purified enzyme after chromatography on the Superdex column was higher (data not shown). This might be due to instability of the recombinant β-mannanase in the buffer at pH 7.5, which was used for equilibration and elution during IMAC chromatography. After purification on the Superdex column as described in the Methods section, the specific activity of the purified enzyme (2570 U mg^-1^) showed a 5.5-fold increase compared with the crude culture supernatant of *P. pastoris *(Table 1).

**Table 1:** One-step purification of recombinant β-mannanase from *A. niger*

Step	Total protein (mg)	Total activity (U)	Specific activity (U mg^-1^)	Degree of purification (fold)	Yield (%)
Culture supernatant	42.6	20870	471	1.0	100
Superdex 75	3.9	10200	2570	5.5	50.7

**Figure 3:**
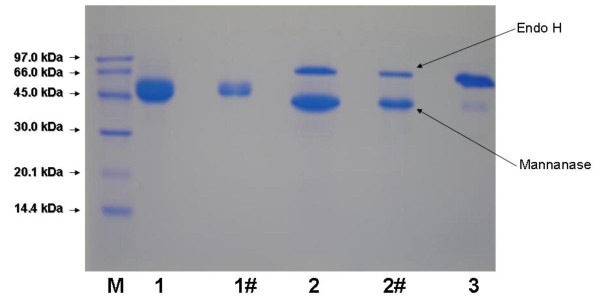
**Deglycosylation of recombinant *A. niger *mannan endo- β-1,4-mannosidase**. SDS-PAGE analysis of the recombinant mannan endo-1,4-β-mannosidase after deglycosylation with endoglycosidase H. Lane M: molecular mass standard protein; Lane 1: purified β-mannanase; Lane 1#: diluted purified β-mannanase; Lane 2: deglycosylated β-mannanase after endoH treatment; Lane 2#: diluted deglycosylated β-mannanase; Lane 3: endoglycosidase H.

Purified recombinant mannan endo-1,4-β-mannosidase displayed one single band with an apparent molecular mass of 53 kDa on SDS-PAGE. When the enzyme was treated with Endoglycosidase H, the apparent molecular mass of the deglycosylated enzyme was approximately 47 kDa (Fig. 3), indicating that mannan endo-1,4-β-mannosidase is a glycoprotein, which is glycosylated to a degree of approximately 12%. IEF revealed one single isoform of pI 4.7 (not shown), in good agreement with the theoretical pI deduced from the amino-acid sequence.

### Characterization of recombinant mannan endo-1,4-β-mannosidase

#### Effect of temperature and pH on enzyme activity and stability

**Figure 4:**
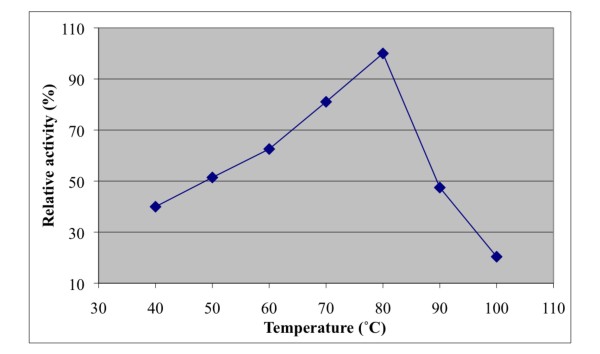
**Effect of temperature on mannan endo- β-1,4-mannosidase activity**. The optimal temperature was determined using 0.5% locust bean gum in 0.1 M citrate-phosphate buffer, pH 4.0, in the 10-min assay.

**Figure 5:**
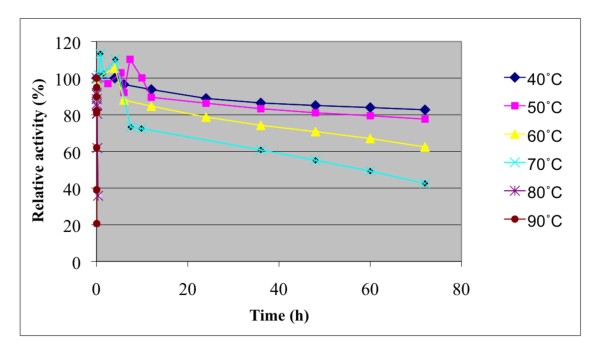
**Effect of temperature on stability of *A. niger* BK01 β-mannanase**. The temperature stability is shown as relative residual activity after incubation without substrate at different temperatures and at pH 4.0.

**Figure 6:**
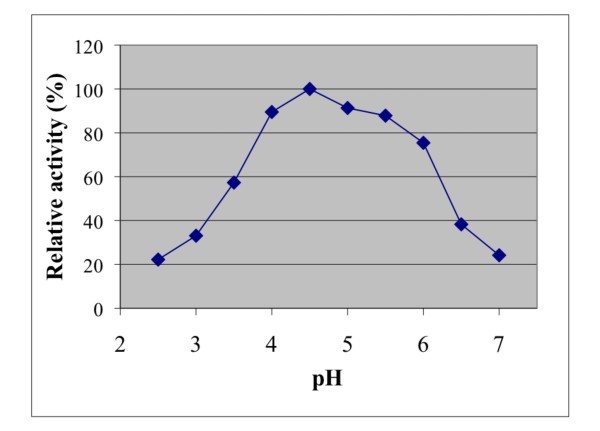
**Effect of pH on mannan endo- β-1,4-mannosidase activity**. The optimal pH was determined at 80°C using 0.5% LBG in 0.1 M citrate-phosphate buffer over a pH range of 2.5-7.0.

**Figure 7:**
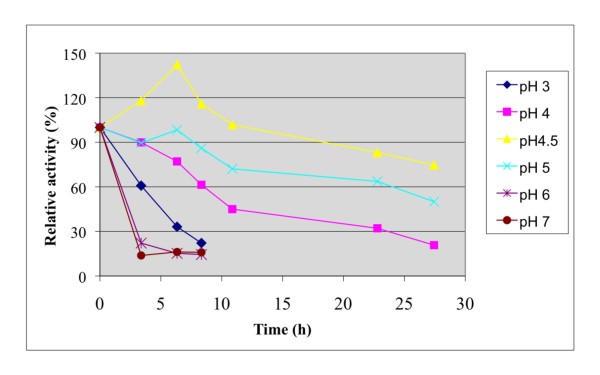
**Effect of pH on stability of *A. niger* BK01 β-mannanase**. The pH stability was reported as relative residual activity after the enzyme was incubated in 0.1 M citrate-phosphate buffer at various pHs without substrate for different periods of time at 50°C.

The effect of temperature and pH were investigated on recombinant *A. niger *BK01 mannan endo-1,4-β-mannosidase using locust bean gum mannan as substrate. The enzyme displayed over 50% activity in the temperature range of 50 to 90°C with an optimal temperature at 80°C for the 10-min assay at pH 6.0 (Fig. 4). The enzyme was stable up to 70°C. Practically all of its activity (>98%) was retained after a 4-h incubation at 70°C, and the half-life time at this temperature was 56 h. At 80°C the half-life time of activity of *A. niger β *-mannanase was only 15 min, and at 90°C about 90% of its activity was lost within 2.5 min of incubation (Fig. 5). The optimal pH for mannan endo-1,4-β-mannosidase (at 80°C) was pH 4.5, and more than 80% of its maximal activity was retained within a rather broad pH range (from pH 4.0-5.5) (Fig. 6). The enzyme was stable in a narrow range around its optimal pH value (pH 4.5-5.0) (Fig. 7)

#### Effect of metal ions and chemical reagents on mannan endo-1,4-β-mannosidase activity

The relative activities (in parenthesis) of the enzyme after incubation with various reagents revealed that mannan endo-1,4-β-mannosidase from *A. niger *BK01 was strongly inhibited by sodium dodecyl sulfate, SDS (20.5%), and Mn^2+ ^(34.9%), and slightly inhibited by phenylmethylsulphonylfluoride, PMSF (75.2%), Na^+ ^(72.6%), Zn^2+^(86.6%), Mg^2+ ^(87.4%), K^+ ^(89.7%), Ca^2+^(90.5%) and Fe^2+^(95.8%). On the contrary, EDTA (235.6%) and Cu^2+ ^(110%) could activate this enzyme.

#### Substrate specificity and kinetics parameters

The relative activity of the purified enzyme on various substrates was determined as described in Methods. Mannan endo-1,4-β-mannosidase from *A. niger *exhibited high activity on locust bean gum (100%), but showed considerably less activity on guar gum (17.9% relative activity) and birchwood xylan (9.1%). Its activity is negligible on starch, carboxymethylcellulose and α-cellulose (<0.1%).

The Michaelis-Menten constants were determined for mannan-containing polysaccharides from various sources (Table 2). These substrates have different structures because of the different ratios of monomers found in the backbone and side chain substituents. For example, glucomannan from konjac has a glucose to mannose ratio of 0.66 to 1; galactomannan from locust bean gum (Sigma) has a mannose to galactose ratio of 4:1, while those from carob (Megazyme) and guar gum have a mannose to galactose ratio of 3.76:1 and 1.6:1, respectively. The K_m _and k_cat _values were 0.6 mg mL^-1 ^and 215 s^-1 ^for low viscosity glucomannan from konjac, 2.0 mg mL^-1 ^and 330 s^-1 ^for locust bean gum galactomannan, 2.2 mg mL^-1 ^and 292 s^-1 ^for low viscosity galactomannan from carob, 1.5 mg mL^-1 ^and 148 s^-1 ^for β-mannan from carob, and 7.7 mg mL^-1 ^and 352 s^-1 ^for guar galactomannan.

**Table 2:** Kinetic parameters for the purified mannan endo-1,4-β-mannosidase^a ^at 70°C

Substrate	V_max_(U mg-1)	K_m_(mg mL-1)	k_cat_(s-1)	kcat/Km(mg^-1 ^s^-1 ^mL)
Glucomannan	243 ± 15	0.6 ± 0.3	215	358
Locust bean gum	373 ± 19	2.0 ± 0.4	330	165
Galactomannan	330 ± 9	2.2 ± 0.2	292	133
Mannan	167 ± 5	1.5 ± 0.2	148	98.4
Guar gum	398 ± 48	7.7 ± 1.8	352	45.7

^a ^Enzymatic reactions were carried out for 5 min at 70°C in 0.07 M citrate phosphate buffer (pH 4.5).

### Product analysis

**Figure 8:**
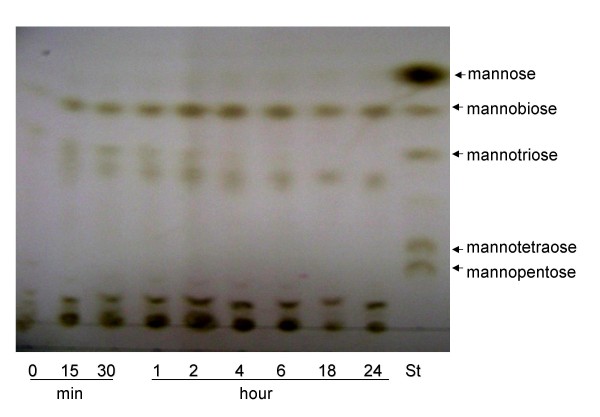
**Thin layer chromatography analysis**. Products from locust bean gum hydrolysis by recombinant *A. niger *mannanase at various time points were separated on silica plates. Incubation times (hour or minute) are indicated. Lane St indicates authentic standards, *i.e*. a mixture of mannose to mannopentaose. Ten μL of hydrolysate was spotted onto each lane.

Analysis of oligosaccharide products obtained during enzymatic hydrolysis of locust bean gum using thin layer chromatography revealed that the recombinant mannan endo-1,4-β-mannosidase from *A. niger *yields mannobiose as its main product, and a small amount of different oligosaccharides (Fig. 8). No trace of mannose could be detected in these hydrolysis experiments.

## Discussion

The mannan endo-1,4-β-mannosidase of *A.niger *BK01 and the hypothetical protein (An05 g01320) of *A.niger *CBS513.18 are highly similar with 99% amino acid sequence identity, and 98% DNA sequence similarity. Their amino acid sequences also match the N-terminal sequence of native *A. niger *mannan endo-1,4-β-mannosidase that was reported in 1998 [24]. We have thoroughly searched the databases and could not find any report on cloning, expression, and characterization of an *A. niger *mannan endo-1,4-β-mannosidase. Thus, our work is the first report on cloning, expression and characterization of this enzyme. In addition, this work also helps verify the function of the hypothetical protein (An05 g01320) of *A.niger *CBS513.18 that has been reported to have strong similarity to mannanase (man1) from *A. aculeatus*. β-Mannanases from *A. niger *BK01 [21], *A. niger *CBS513.18 and *A. niger *published by Ademark et al [24] appear to be closely related and have similar properties, which are superior than those from *A. niger *NRRL 337 [25]. Mannan endo-1,4-β-mannosidases from other *Aspergillus species *e.g., those from *A. aculeatus, A. fumigatus *or *A. sulphureus*, have previously been cloned and expressed in various hosts [26-31]. *A. niger *β-mannanase has unique properties that are different from other *Aspergillus *mannanases. Amino acid sequence of *A. niger *BK01 mannan endo-1,4-β-mannosidases including putative signal peptide can be found in additional file 1.

Mannan endo-1,4-β-mannosidases or β-mannanases have been classified into two family of glycoside hydrolases, *i.e*., family 5 and 26, based on their sequence similarity http://www.cazy.org. The *A. niger *mannan endo-1,4-β-mannosidase belongs to glycoside hydrolase family 5 (GH5). Analysis of the primary sequence of fungal mannan endo-1,4-β-mannosidases belonging to this family revealed amino acid sequence similarities ranging from 43 to 93%. Some fungal β-mannanases have been shown to contain a cellulose-binding domain (CBD) at either the C-terminus or N-terminus of the enzyme. For example, CBD of *T. reesei β *-mannanase is located at its C-terminus (amino acid position 373-410) preceded by a serine-, threonine-, and proline-rich region [29], whereas CBD of *A. fumigatus *β-mannanase is located at its N-terminus (amino acid position 9-44) as shown in Fig. 1. A role of this domain in the hydrolysis of mannan/cellulose complex substrates has been suggested [32]. However, β-mannanases from different fungi including *A. niger *do not contain a CBD. The protruding N-terminus of *Bispora sp*. as shown in the sequence alignment in Fig. 1 is not similar to a CBD, and its role in catalysis still remains to be explored.

The mature *A. niger *mannan endo-1,4-β-mannosidase gene without its signal peptide was expressed in *P. pastoris *X33 with the C-terminus of the recombinant enzyme fused to the 6xHis tag for affinity purification by immobilized metal affinity chromatography (IMAC). The native signal peptide was replaced with that of the *Saccharomyces cerevisiae *α-factor, allowing secretion of the active enzyme into the extracellular medium with an expression yield of approximately 243 mg L^-1^. Since cultivation experiments were done in shake flasks, one can expect that growth of *P. pastoris *in a fermentor under controlled and optimised conditions will result in considerably higher protein yields [33]. In comparison, the expression level of *Trichoderma reesei *mannan endo-1,4-β-mannosidase in *S. cerevisiae *was roughly 150 mg L^-1 ^[29] and that of *Bispora *sp. MEY-1 [15] and *B. subtilis *mannan endo-1,4-β-mannosidase in *P. pastoris *GS115 [34] was 1800 mg L^-1 ^and 150 mg L^-1^, respectively. Moreover, the enzyme activity during fermentation reached 670 U mL^-1^, which is significantly higher than those reported for most mannan endo-1,4-β-mannosidases [26,34].

Amino acid sequence analysis by the NetNGlyc 1.0 sever program indicated two putative N-glycosylation sites in the amino acid sequence of mannan endo-1,4-β-mannosidase *i.e*., ^195^NSS^197^, and ^252^NFT^254 ^(according to numbering in Fig. 1). The deglycosylation analysis, showed in Fig. 3, confirms the prediction that the enzyme is glycosylated. Purified mannan endo-1,4-β-mannosidase treated with Endoglycosidase H was calculated to be 43 kDa by SDS-PAGE. This result indicated that the *A. niger *mannan endo-1,4-β-mannosidase expressed in *P. pastoris *was properly glycosylated, as the enzyme was as active as the native enzyme.

Recombinant *A. niger *BK01 mannan endo-1,4-β-mannosidase efficiently hydrolysed galactomannans, glucomannans and β-1,4-mannans from different sources. Although determination of kinetic parameters enabled us to demonstrate that *A. niger *GH5 mannan endo-1,4-β-mannosidase was highly active towards structurally different mannans, this enzyme displayed highest specificity towards unsubstituted carob glucomannan with a K_m _and k_cat _of 0.6 mg.mL^-1 ^and 215 s^-1^, respectively. Based on these kinetic parameters, mannanase activity of this enzyme appears not to be hampered by the presence of side chains, *i.e*. galactosyl residues. The enzyme can hydrolyse galactomannans as well as glucomannans and unsubstituted β-1,4-mannan. The action of the enzyme on these substrates as well as on Azo-carob galactomannan, a standard substrate for the assessment of mannan endo-1,4-β-mannosidase activity, indicates its function as a true endo-β-1,4-mannanase. This activity was confirmed by TLC analysis of reaction products obtained through hydrolysis of locust bean gum, which gave mannobiose as major product and no detectable mannose, indicating that the enzyme has no β-mannosidase activity. In addition to mannan, *A. niger *mannanase was capable of degrading birchwood xylan (9.1% relative activity). Its lacks of activity for cellulose is valuable for applications in bleaching pulp and paper [1].

In general, the properties of the recombinant mannan endo-1,4-β-mannosidase from *A. niger *are very similar to those of the enzyme from its natural source with only some minor differences. The recombinant *A. niger *mannan endo-1,4-β-mannosidase furthermore shows properties typical of thermostable enzymes, which is in accordance with the wild-type enzyme. The optimal temperature of its activity is 80°C, similar to that of the native enzyme [21]. Optimal temperatures of different fungal β-mannanases have previously been reported; that of the β-mannanase from *Trichoderma reesei *C-30 was found at 75°C [35], whereas β-mannanase produced from *A. niger *and *A. flavus *showed their optimum at 65 and 60°C, respectively [14]. Two forms of mannan endo-1,4-β-mannosidase from the thermotolerant fungus *A. fumigatus *IMI 385708 showed highest activity at 60°C (pH 4.5) [6]. These rather high optima appear to be a common but valuable characteristic of fungal β-mannanases. At such high temperatures (above 60-65°C), enzymatic digestion may not only increase the rate of hydrolysis but also reduce microbial contamination of the material being processed [36].

Both native and recombinant *A. niger *mannan endo-1,4-β-mannosidase appear to be among the most thermostable fungal β-mannanases reported to date. The recombinant *A. niger *mannan endo-1,4-β-mannosidase retained >98% activity after 4 h at 70°C, while mannan endo-1,4-β-mannosidase from *Talaromyces emersonii *and *Aspergillus niger *NRRL 337 retained 53% and 20% activity after 1 h at 65°C, respectively [36]. The half-life of thermostable acidic mannan endo-1,4-β-mannosidase from *Sclerotium (Athelia) rolfsii *at 70°C and pH 4.5 was 1.5 h [37], while that of recombinant *A. niger *mannan endo-1,4-β-mannosidase at 70°C was 56 h. *Bispora *sp. MEY-1 mannan endo-1,4-β-mannosidase, which was expressed in *P. pastoris*, only retained more than 50% of its initial activity after an incubation at 70°C for 20 min [15].

The strong increase of the enzyme activity in the present of EDTA (1 mM) suggests that metal ions are not present in the active site and are not required for activity. At the same concentration, the effect of EDTA on *A. sulphureus *β-mannanase activity was only moderate [26]. EDTA might protect the enzyme against the detrimental effect of metal ions present in the enzyme preparation, which inhibit the *A. niger *β-mannanase to a certain extent while not affecting the *A. sulphureus *enzyme significantly. For example, Mn^2+ ^strongly inhibited the *A. niger β *-mannanase but did not effect the latter enzyme. Other ions including Na^+^, Zn^2+^, Mg^2+^, Ca^2+^, Fe^2+ ^exerted an adverse effect on the activity of *A. niger *β-mannanase but did not affect or slightly increased the *A. sulphureus *β-mannanase activity [26]. The β-mannanase activity inhibition in the presence of PMSF (1 mM) was 24.8%, suggesting the role of a serine in the catalytic action of *A. niger *mannan endo-1,4-β-mannosidase.

## Conclusion

To date, this study is the first report on production of a thermostable GH5 mannan endo-1,4-β-mannosidase from *A. niger *using a *P. pastoris *expression system. This kind of thermostable enzyme can gain a great deal of interest for industrial applications in large scale due to an increasing demand towards renewable resource utilization in the near future.

## Methods

### Strains, plasmids, enzymes, reagents, and growth media

*Aspergillus niger *strain BK01, isolated from an orange in Vietnam, was identified by the Center of Biotechnology, Vietnam National University, Hanoi, and is preserved in our laboratory. *Escherichia coli *strain TOP 10 (Invitrogen) was used as a host for molecular cloning of DNA in pPICZαA (Invitrogen) and propagation of recombinant expression vectors. *Pichia pastoris *X33 (Invitrogen) was used for heterologous protein expression. All media and protocols for *Pichia *are described in the *Pichia *expression manual (Invitrogen). Manno-oligosaccharides were from Megazyme (Bray, Ireland).

### Total RNA isolation, cDNA synthesis and cloning of the mannan endo-1,4-*β*-mannosidase gene

*A. niger *BK01 was grown at 37°C for 48 h in a plate containing 10 mL liquid modified Czapek medium including locust bean gum as a sole carbon source (5 g L^-1^). The mycelium was then transferred to a plate containing 10 mL β-mannanase induction medium based on modified coffee pulp waste (21.6 g L^-1^) and guar gum (10 g L^-1^) [21]. After a 72-h growth period, the mycelium was harvested. Total RNA was isolated by extracting 50 mg of mycelium using the RNeasy Plant Mini Kit (Qiagen, Valencia, CA). cDNA was synthesized from RNA using One Step RT-PCR Kit (Qiagen) according to the manufacturer's instructions. The primers used were CF (5'-ATG AAG CTT TCC AAC GCC CTC CTC-3') and CR (5'-TTA AGC ACT ACC AAT AGC AGC AAC ATG ATC-3'), which were designed according to the amino acid sequence of the putative mannan endo-1,4-β-mannosidase from *A. niger *CBS 513.88 (Genbank accession no. XP_001390707) The thermocycling parameters were: 50°C for 35 min, 95°C for 15 min and 30 cycles of 94°C - 30 s, 54°C - 45 s and 72°C - 90 s, followed by 10 min extension at 72°C.

To subclone the mannan endo-1,4-β-mannosidase cDNA into the expression vector, the β-mannanase-encoding sequence was amplified from the previously isolated cDNA with the primers PNf (5'-CTG TGC GAA TTC TCC TTC GCC AGC ACC TCC G-3'), including an *EcoR*I site, and PRv (5'-CTG TGC TCT AGA GCA CTA CCA ATA GCA GCA ACA TGA TCC -3'), including a *Xba*I site, using a mixture of *Pfu *DNA polymerase (3 U/reaction) and *Taq *DNA polymerase (3 U/reaction). The thermocycling parameters were 95°C for 2 min, and 30 cycles of 95°C for 45 s, 59°C - 45 s and 72°C - 3 min. The DNA insert was cloned into *EcoR*I and *Xba*I sites of the pPICZαA vector, downstream of the α-factor signal peptide sequence. Proper construction was confirmed by restriction digestion and automated DNA sequencing (Macrogen Inc., Korea). The DNA sequence encoding mature mannan endo-1,4-β-mannosidase was deposited in the GenBank database under accession no. FJ268574.

### Expression of the mannan endo-1,4-*β*-mannosidase gene in *Pichia pastoris*

The selected expression plasmid was linearized with *Sac*I (New England BioLabs, USA), and then transformed into *P. pastoris *strain X33 by electroporation [22]. Transformants were first screened from YPDS (1% yeast extract, 2% peptone, 2% dextrose, 1 M sorbitol, 2% agar) plates containing Zeocin™ at a final concentration of 100 μg mL^-1^, then on YPDZ plates (1% yeast extract, 2% peptone, 2% dextrose and 2% agar containing Zeocin at final concentrations of 150, 300, and 500 μg mL^-1^) in order to screen for higher copy numbers of the targeted gene. Recombinant strains producing mannan endo-1,4-β-mannosidase were further confirmed by BMGY-Azo plates (1% yeast extract, 2% peptone, 100 mM potassium phosphate pH 6.0, 1.34% YNB, 4 × 10^-5 ^% biotin, 0.5% methanol, 2% agar and 0.3% Azo-carob galactomannan). Mut^+ ^phenotype of *P. pastoris *recombinants was analysed by genomic PCR with 5'AOX and 3'AOX primers. The culture medium from the *P. pastoris *transformant that had the highest β-mannanase activity was used for the subsequent analysis of the recombinant protein. A control was transformed with an empty pPICZαA plasmid.

### Purification of recombinant mannan endo-1,4-*β*-mannosidase

All purification steps were performed at 4°C unless stated otherwise. The crude culture supernatant was obtained by centrifugation of the culture broth at 4000 rpm for 5 min at 4°C. The crude supernatant was concentrated 30-fold by ultrafiltration through a 10-kDa cut-off membrane (Sartorius Stedim Biotech, Germany). The final purification was done by size exclusion chromatography on a Superdex 75 HR 10/30 column (Amersham Pharmacia Biotech, Sweden) equilibrated with 50 mM potassium phosphate buffer, pH 6.0, and the protein was eluted at a flow rate of 1 mL min^-1^. Active fractions were combined and used in further experiments as the purified β-mannanase.

### Polyacrylamide gel electrophoresis and zymograms

Sodium dodecyl sulfate polyacrylamide gel electrophoresis (SDS-PAGE) was performed in a 12.5% (w v^-1^) polyacrylamide gel by the method of Laemmli (1970). Proteins were stained with Coomassie brilliant blue G-250.

Zymograms were obtained by co-polymerizing 0.2% (w v^-1^) locust bean gum with 12.5% (w v^-1^) polyacrylamide gel as previously reported [38]. After electrophoresis, the gel was soaked in 2.5% cold Triton X100 with gentle shaking to remove SDS and re-fold the proteins in the gel. The gel was then washed four times at 4°C in 50 mM potassium phosphate buffer pH 6.0 for 30 min. After incubation for 45 min at 50°C, the gels were stained with Congo red solution (0.1%, w v^-1^), and destained with 1 M NaCl. The activity bands were observed as clear yellow halos.

Deglycosylation of recombinant β-mannanase Purified mannan endo-1,4-β-mannosidase was deglycosylated by denaturing the protein at 100°C for 10 min prior to the addition of endoglycosidase H_f _(Endo H_f_, New England Biolabs) at 37°C for 1 h according to the manufacturer's instructions.

### β-Mannanase assay and protein quantification

Mannan endo-1,4-β-mannosidase activity was determined using the 3,5-dinitrosalicylic acid (DNS) method [39]. The reaction was started by mixing 0.1 mL of appropriately diluted enzyme sample with 0.9 mL of 5 mg mL^-1 ^locust bean gum in 0.1 M citrate-phosphate buffer, pH 4.0. After 10-min incubation at 50°C, the reaction was stopped by the addition of 5 mL DNS reagent. One unit of mannan endo-1,4-β-mannosidase activity is defined as the amount of enzyme releasing 1 μmole of mannose equivalents per minute. All experiments were done in triplicate, and average values are reported. Protein concentration was determined with bovine serum albumin as standard, using the Micro-BCA Protein Assay Reagent (Biorad, USA).

### Effect of temperature, pH and various reagents on enzyme activity

The optimal temperature for mannan endo-1,4-β-mannosidase activity (10-min assay) was determined by the standard activity assay as described above at various temperatures ranging from 40 to 100°C. To estimate thermal stability, the enzyme was pre-incubated in 0.1 M citrate-phosphate buffer (pH 4.0) at various temperatures (60-90°C) for different periods of time without substrate and then assayed for residual activity at 50°C using the standard activity assay. The effect of pH on β-mannanase activity was determined at 80°C over a pH range of 2.5-7.0 by using 0.1 M citrate-phosphate buffer. To estimate pH stability, the enzyme was diluted/pre-incubated in the same buffer at different pH-values for different periods of time at 50°C without substrate, and then assayed for residual activity at pH 4.0. The effect of ions on mannan endo-1,4-β-mannosidase activity was examined by incubating the enzyme with 1 mM of various metal ions in 0.1 M sodium citrate buffer (pH 4.0) at 50°C for 30 min.

### Substrate specificity and kinetic parameters

The substrate specificity of purified recombinant mannan endo-1,4-β-mannosidase was evaluated with the following substrates at 5 mg mL^-1 ^in 0.1 M citrate-phosphate buffer, pH 4.0: locust bean gum (Sigma), guar gum (Sigma), birchwood xylan (Roth), soluble starch (Sigma-Aldrich), α-cellulose and carboxyl methyl cellulose (Fluka). Activity was measured by the dinitrosalicylic acid (DNS) method as described above. For the kinetic experiments, 0.1 M citrate-phosphate buffer (pH 4.5) containing 0.1 to 10 mg mL^-1 ^of various mannan substrates (*i.e*., locust bean gum (Sigma), guar gum (Sigma), carob galactomannan (low viscosity) (Megazyme), konjac glucomannan (low viscosity) (Megazyme), and carob 1,4-β-D-mannan (Megazyme), was incubated with the purified mannan endo-1,4-β-mannosidase at 70°C for 5 min. Kinetic parameters (K_m _and v_max_) were calculated from the experimentally obtained data and non-linear regression analysis using the "GraFit" software (Erithacus Software Ltd.).

### Thin layer chromatography

Locust bean gum was prepared at a concentration of 1% (w v^-1^) in 50 mM sodium citrate buffer, pH 4.5. After addition of the purified enzyme (7.5 U for 2-mL reactions), the solution was incubated at 40°C. Aliquots were removed at various time, and heated to 100°C for 10 min. Hydrolysis products were separated on silica plates 60 F 254 (Merck, Darmstadt, Germany) using a solvent system consisting of 1-propanol-nitromethane-water (7:1:2, v v^-1^). The products were detected by spraying with 5% sulphuric acid in ethanol followed by heating at 110°C for about 5 min. Manno-oligosaccharides (mannose, mannobiose, mannotriose, mannotetraose and mannopentose, Megazyme) were used as standards.

## Competing interests

The authors declare that they have no competing interests.

## Authors' contributions

DBC performed most parts of the experiments and prepared the first draft of the manuscript. DTT and TKA directed the study. JBG checked all data, designed the frame of this publication and revised the draft. JCS co-designed the frame of this publication. DTT, TKA, JGB and JCS also co-supervised enzyme characterization. DH initiated expression in *Pichia pastoris *and edited the manuscript. MY conceived of the study, supervised molecular biology work, participated in sequence alignment and edited the manuscript. All authors read and approved the final manuscript.

## Supplementary Material

Additional file 1 **Complete amino acid sequence of *A. niger *BK01 mannan endo-1,4-β mannosidase including native signal peptide**. Amino acid sequence in FASTA format of the entire mannan endo-1,4-β-mannosidase from *Aspergillus niger *BK01, including putative signal peptide. Click here for file
