# Supplementary material for: Cloning, expression in Pichia pastoris, and characterization of a thermostable GH5 mannan endo-1,4-β-mannosidase from Aspergillus niger BK01
Source: Microb Cell Fact. 2009 Nov 13;8:59. doi: 10.1186/1475-2859-8-59 (PMC2780388; doi:10.1186/1475-2859-8-59)
Supplement: Additional file 1 — Complete amino acid sequence of A. niger BK01 mannan endo-1,4-β mannosidase including native signal peptide. Amino acid sequence in FASTA format of the entire mannan endo-1,4-β-mannosidase from Aspergillus niger BK01, including putative signal peptide. [file 1475-2859-8-59-S1.pdf]

>A.nigerBK01 Mannanase
MKLSNALLTLASLALANVSTALPKASPAPSTSSSAASTSFASTSGLQFTIDGETGYFAGTNSYWIGFLTDNADVDL
VMGHLKSSGLKILRVWGFNDVTSQPSSGTVWYQLHQDGKSTINTGADGLQRLDYVVSSAEQHDIKLIINFVNYWTD
YGGMSAYVSAYGGSGETDFYTSDTMQSAYQTYIKTVVERYSNSSAVFAWELANEPRCPSCDTSVLYNWIEKTSKFI
KGLDADRMVCIGDEGFGLNIDSDGSYPYQFSEGLNFTMNLDIDTIDFGTLHLYPDSWGTSDDWGNGWITAHGAACK
AAGKPCLLEEYGVTSNHCSVEGAWQKTALSTTGVGADLFWQYGDDLSTGKSPDDGNTIYYGTSDYQCLVTDHVAAI
GSA
